# Functionality and stability data of detergent purified nAChR from *Torpedo* using lipidic matrixes and macroscopic electrophysiology

**DOI:** 10.1016/j.dib.2015.12.010

**Published:** 2015-12-25

**Authors:** Luis F. Padilla-Morales, José O. Colón-Sáez, Joel E. González-Nieves, Orestes Quesada-González, José A. Lasalde-Dominicci

**Affiliations:** a Department of Chemistry, University of Puerto Rico, Río Piedras Campus, San Juan, PR, United States; b Department of Pharmaceutical Sciences, School of Pharmacy, Medical Sciences Campus University of Puerto Rico, San Juan, PR, United States; c Department of Biology, University of Puerto Rico, Río Piedras Campus, San Juan, PR, United States; d Department of Physical Sciences, University of Puerto Rico, Río Piedras Campus, San Juan, PR, United States; e Molecular Science Building, University of Puerto Rico, San Juan, PR, United States

**Keywords:** Detergents, Fluorescence recovery after photobleaching, Lipidic Cubic Phase, nAChR, Planar lipid bilayer, Two-electrode voltage clamp

## Abstract

The presented data provides additional information about the assessment of affinity purified nicotinic acetylcholine receptor (nAChR) rich membrane solubilized with long chain (16 saturated carbons) lysophospholipid with glycerol headgroup (LFG-16). The assessment of stability and functionality of solubilized membrane protein is a critical step prior to further crystallization trails. One of the key factors for this task is the appropriate choice of a detergent that can support nAChR activity and stability comparable to the crude membranes. The stability of the nAChR-LFG-16 complex incorporated into lipid cubic phase (LCP) was monitored for a period of 30 days by means of fluorescence recovery after photobleaching (FRAP) and the functionality was evaluated after its incorporation into *Xenopus oocyte* by means of the two electrode voltage clamp technique.

**Specifications Table**

**Table:** 

Subject area	*Biochemistry*
More specific subject area	*Membrane protein, oocyte electrophysiology*
Type of data	*Graph and figure*
How data was acquired	*Two electrode voltage clamp and FRAP Assay using a Zeiss LSM 510 confocal microscope*
Data format	*Filtered and analyzed*
Experimental factors	*Application of lipid analogue detergent*
Experimental features	The stability and functionality of solubilized nAChR was examined by fluorescence recovery after photobleaching and two electrode voltage clamp techniques
Data source location	N/A
Data accessibility	*Data is supplied in this article*

**Value of the data**

• The unique approach used to assess functional activity of an ion channel-detergent complex provides a practical and rapid method for screening activity of other membrane protein detergent complex prior to crystallization trials.
• The result provided here may forewarn some researchers who are using traditional detergent for the solubilization of membrane protein about the possible effects of detergent structure on channel functionality.
• The data can be useful for other researchers investigating the effects of different detergent head groups on the stability of solubilized membrane proteins.

## 1 Data

**Fig. 1:**
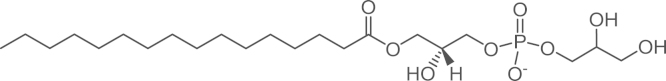
Structure of the phospholipid analog detergents 1-palmitoyl-2-hydroxy-*sn*-glycero-3-phospho-(1′-*rac*-glycerol) (LFG-16) used for the solubilization nicotinic acetylcholine receptor from *Torpedo californica* electric organ, using the phospholipid analog detergent 1-palmitoyl-2-hydroxy-*sn*-glycero-3-phospho-(1′-*rac*-glycerol) (LFG-16).

**Fig. 2:**
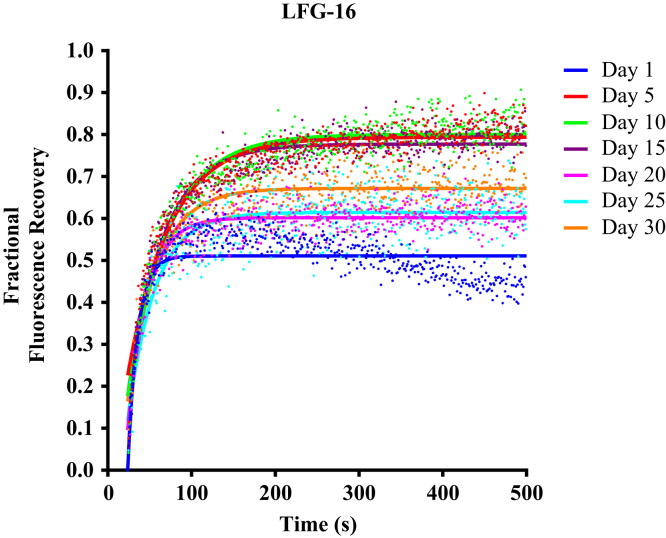
Phospholipid analog detergents lipidic matrix stability, LCP-FRAP assay. Fractional fluorescence recovery and diffusion coefficient of each affinity purified nAChR using the phospholipid analog detergent 1-palmitoyl-2-hydroxy-*sn*-glycero-3-phospho-(1′-*rac*-glycerol) (LFG-16). FRAP experiments were recorded every five days for 30 days. All fluorescence recovery experiments were performed in triplicates, averaging five recoveries on different areas of the lipidic matrix with the nAChR incorporated. The fractional recovery was calculated using equation$F\left(t\right)=\left[\frac{f_{t}-f_{0}}{f_{\infty }-f_{0}}\right]$ where *f*_(*t*)_ is the corrected fluorescence intensity of the bleached spot, *f_0_* is the corrected fluorescence intensity of the bleached spot in the 600 msec after bleaching, and $f_{\infty }$ is the average of corrected fluorescence intensity in the five pre-bleached images.

We provide additional data about the stability and functionality of nAChR solubilized from *Torpedo californica* with the lipid analog detergent, 1-hexadecanoyl-*sn*-glycero-3-phospho-(1′-*rac*-glycerol) (LFG-16). The stability of the affinity purified nAChR-LFG-16 detergent complex was determined after it incorporation into lipid cubic phase (LCP) of 1-(cis-9-Octadecenoyl)-rac-glycero for a period of 30 days using Fluorescence Recovery after Photobleaching (FRAP)(Fig. 1). The functionality of the purified nAChR-LFG-16 detergent complex was studied after reconstitution into *Xenopus* oocyte by mean of two electrode voltage clamp (Fig. 2).

## 2 Experimental design, materials and methods

### 2.1 Crude membrane protocol

**Fig. 3:**
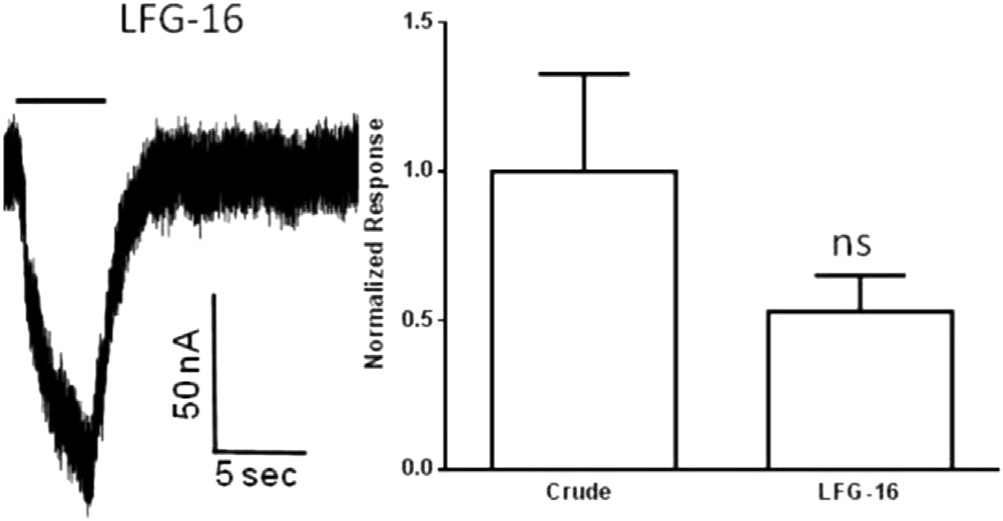
Macroscopic ion channel functional assay of LFG-16 solubilized and affinity purified nAChR-DCs. Responses were evoked by a 5 second application of 100 μM ACh (represented by bars) at −70 mV on *Xenopus* oocytes injected with LFG-16 solubilized purified nAChR-DCs. Responses were normalized to the respective crude membranes used for solubilization plotted as mean ±SEM and compared using an unpaired *t*-test in Graph Pad Prism 6.

nAChR extraction was performed homogenizing 60 g of *Torpedo californica* tissue for 4 min in cold room with 120 ml of buffer A (100 mM NaCl, 10 mM Sodium Phosphate, 5 mM EDTA, 5 mM EGTA, 5 mM DTPA, 0.02% Sodium Azide, pH 7.4) mixed with 120 μl of phenyl methane sulfonyl fluoride (PMSF) and 0.112 g of Iodoacetamide. The mixture of buffer and tissue was homogenized using a blender on high-liquefy for 4 min. The homogenate was transferred to centrifuge tubes and centrifuged for 30 min at 6500 rpm at 4 °C. The supernatant was filtered through gauze and centrifuged for 30 min at 40,000 rpmat 4 °C. Consequently, the pellet from this spin was resuspended in 100 mL Buffer B (10 mM Sodium Phosphate, 5 mM EDTA, 5 mM EGTA, 5 mM DTPA, 0.02% Sodium Azide, pH 7.8) mixed with 100 μL PMSF. This mixture was once again spun for 30 min at 6000 rpm at 4 °C. The supernatant from this spin was centrifuged for 30 min at 40,000 rpm at 4 °C. The pellet was resuspended in 25 mL of 40% sucrose storage solution and these crude membranes were properly labeled and stored at −80 °C until ready to use (Fig. 3).

### 2.2 Affinity column purification of solubilized nAChR

All steps were carried out in the cold room or on ice. In order to solubilize the crude membranes, these were thawed and mixed with a 10% (w/v) detergent solution and DB-1X Buffer (100 mM NaCl, 10 mM MOPS, 0.1 mM EDTA, 0.02% NaN_3_) for a final concentration of detergent 1–4%. The DB-1X buffer was added first, followed by the detergent and finally the crude membranes, which were added drop by drop. This solution was shaken slowly for 1 h and then centrifuged at for 1 h at 40krpm and 4 °C. The supernatant was extracted and used immediately for the affinity-column purification. Approximately 12 mL of previously prepared bromoacetylcholine affinity resin (Bio-Rad Laboratories, Hercules, CA) in a 1.5×15 cm Econocolumn (Bio-Rad Laboratories, Hercules, CA) was drained of storage buffer (40% Sucrose, 2 mM PMSF) was conditioned with 50 mL of ddH_2_O and 50 mL of 1.5 CMC detergent buffer before the supernatant prepared previously was added to the column. The column was washed with 50 mL of 1.5 CMC detergent buffer (Fig. 1) before the nAChR was eluted with 50 mL of elution buffer. The sample was then concentrated using centrifuge filter with a 100 K cutoff (Amicon Ultra Centrifugal Filters Ultracel 100 K, Millipore Co., Billerica, MA)) and run through a P-10 desalting column (GE Healthcare, Uppsala, Sweden) to remove the carbamylcholine ligand. Our sample was eluted with 5 mL of 1.5 CMC detergent buffer and finally concentrated to 250 μL. Protein concentration was determined using a BCA Protein Concentration Assay (Pierce biotechnology, Rockford, IL) and a sodium dodecyl sulfate polyacrylamide gel electrophoresis (SDS-PAGE) was run to verify receptor purity.

### 2.3 FRAP assays

FRAP experiments were performed according to the conditions and protocols described by Cherezov et al. (2008) [7], with the following modifications [2], [3]: 50 μl of a solution containing 2.0 mg/ml of ligand-affinity purified nAChR was incubated with (α-BTX) conjugated with Alexa-488 (Invitrogen, Carlsbad, CA) in a 1:2.5 ratio for 1.5–2 h in the dark at 4 °C. The nAChR-detergent-α-BTX complex was mixed with molten monoolein in a 2:3 volume ratio, using a syringe lipid mixer, and mixed until it was completely clear. The nAChR-detergent-α-BTX complex in LCP was placed on a 75×25 mm slides and washed with 1.5 ml of 1.5 CMC detergent buffer solution three times before recovering the LCP-nAChR-detergent-α-BTX complex with a syringe. The LCP- nAChR-detergent-α-BTX was transferred into an automatic sampler, and approximately 0.2 μl of LCP- nAChR-detergent-α-BTX was dispensed into 7 mm diameter wells formed by punching holes into 50 lm thick transfer tape (9482 PC; 3 M, Minneapolis, MN) and pressing onto a glass slide. The LCP-FRAP wells were covered immediately by pressing a coverslip against the slide and flattening with a rubber roll [6]. This procedure was performed quickly to form a tight seal; otherwise, the LCP could dry out and compromise matrix integrity. The entire experimental procedure was performed in an environment with a relative humidity range of 60–80%.

### 2.4 FRAP Instrument setup and data collection

All FRAP data was collected 24 h after plates were assembled. Data collection for FRAP assays was performed at room temperature using a Zeiss (Thornwood, NY) LSM 510 confocal microscope with an objective of ×40. Five pre-bleach images were used to establish baseline fluorescence, and the laser was triggered to bleach at 75% power, immediately followed by a sequence of 500 images scanning at 2.6% power with a 0.6-s laser scanning delay. All images were obtained and processed using the Zeiss ZEN software. For data analysis each sample was integrated within a 14.0-μm-diameter circular region of interest (ROI_1_). Averaged integrated intensity of another 14.0-μm circular region of interest (ROI_2_), positioned near the bleached ROI_1_, was used to correct for photobleaching from irradiation during the image-acquisition sequence. Fluorescence intensity was corrected by dividing the value of the integrated intensity ROI_1_ in the bleached spot by the average integrated intensity of the ROI_2_. As described by Cherezov et al. [7]. The fractional fluorescence recovery curves, *F*(*t*), were calculated according [1].

### 2.5 Injection of oocytes with crude or nAChR detergent complex and two electrode voltage clamp assays

We used a modified version of the protocol originally used by the Miledi and Morales research group [4], [5]. *The Xenopus laevis* oocyte were obtained by surgical extraction, defolliculated and the selected oocyte were microinjected with with 50 nL of 6 mg/mL of crude membrane or 3 mg/mL of 1.5 fold critical micellar concentration nAChR detergent complex, affinity purified from *Tc*. In Padilla et al. 2015 we provide a complete description of both protocols [1] (Fig. 3).
